# An immune-modulating diet increases the regulatory T cells and reduces T helper 1 inflammatory response in Leishmaniosis affected dogs treated with standard therapy

**DOI:** 10.1186/s12917-015-0610-7

**Published:** 2015-12-03

**Authors:** Laura Cortese, Mariangela Annunziatella, Anna Teresa Palatucci, Sarah Lanzilli, Valentina Rubino, Alessandro Di Cerbo, Sara Centenaro, Gianandrea Guidetti, Sergio Canello, Giuseppe Terrazzano

**Affiliations:** Department of Veterinary Medicine and Animal Productions, Division of Internal Medicine, University of Naples Federico II, Via Delpino, 1, 80137 Naples, Italy; School of Science, University of Basilicata, Via Sauro, 85, 85100 Potenza, Italy; Department of Translational Medical Sciences, University of Naples Federico II, Via Pansini, 5, 80131 Naples, Italy; School of Specialization in Clinical Biochemistry, “G. d’Annunzio” University, Chieti, Italy; Division of Research and Development, Sanypet SpA, Padova, Italy; Department of Science, University of Basilicata, Via Sauro, 85, 85100 Potenza, Italy

**Keywords:** Leishmaniosis, Pharmacological treatment, Nutraceuticals, Nutrition, Immune profile, Treg

## Abstract

**Background:**

Clinical appearance and evolution of Canine Leishmaniosis (CL) are the consequence of complex interactions between the parasite and the genetic and immunological backgrounds. We investigated the effect of an immune-modulating diet in CL. Dogs were treated with anti- *Leishmania* pharmacological therapy combined with standard diet (SD Group) or with the immune-modulating diet (IMMD Group). CD3+ CD4+ Foxp3+ Regulatory T cells (Treg) and CD3+ CD4+ IFN-γ + T helper 1 (Th1) were analyzed by flow cytometry.

**Results:**

All sick dogs showed low platelet number at diagnosis (T0). A platelet increase was observed after six months (T6) SD Group, with still remaining in the normal range at twelve months (T12). IMMD Group showed an increase in platelet number becoming similar to healthy dogs at T6 and T12. An increase of CD4/CD8 ratio was revealed in SD Group after three months (T3), while at T6 and at T12 the values resembled to T0. The increase in CD4/CD8 ratio at T3 was maintained at T6 and T12 in IMMD Group.

A reduction in the percentage of Treg of all sick dogs was observed at T0. A recovery of Treg percentage was observed only at T3 in SD Group, while this effect disappeared at T6 and T12. In contrast, Treg percentage became similar to healthy animals in IMDD Group at T3, T6 and T12. Sick dogs showed an increase of Th1 cells at T0 as compared with healthy dogs. We observed the occurrence of a decrease of Th1 cells from T3 to T12 in SD Group, although a trend of increase was observed at T6 and T12. At variance, IMMD Group dogs showed a progressive decrease of Th1 cells, whose levels became similar to healthy controls at T6 and T12.

**Conclusion:**

The immune-modulating diet appears to regulate the immune response in CL during the standard pharmacological treatment. The presence of nutraceuticals in the diet correlates with the decrease of Th1 cells and with the increase of Treg in sick dogs. Therefore, the administration of the specific dietary supplement improved the clinical response to the standard treatment in a model of CL.

## Background

Canine Leishmaniosis (CL) is a zoonotic disease for humans and dogs and is caused by the protozoan parasite *Leishmania* (*L.*) *infantum* in the Mediterranean area [1].

Several clinical manifestations have been described in CL [2, 3] and the clinical appearance and evolution of Leishmaniosis appear to be the consequence of complex interactions between the parasite and the genetic and immunological profile of the host [1, 4]. CL is a non self-limiting infection causing severe disease [1–3], but is often manifested as sub clinical infection with the features of a self-limiting disease [5, 6].

Peculiar immunological profiles characterize the two opposite extremes of this clinical spectrum: the cell-mediated immunity, mainly based on Interferon (IFN)-γ secreting T helper (Th) 1 lymphocytes, and the anti-*Leishmania* macrophage activity, which has been associated with self-limiting disease [7].

In contrast, occurrence of severe illness has been described in presence of a marked humoral immune response, accompanied by reduced or depressed cell mediated immunity with mixed Th1 and Th2 cytokine responses [1, 7]. Clinical signs of disease range from a mild dermatitis and alopecia, associated with specific cellular immunity [8], to a severe disease with renal damage and glomerulonephritis [9]. *L. infantum* infected dogs could remain clinically healthy for an indeterminate period of time or life along [10]. Such occurrence has been associated with the cellular Th1 immunity [1, 11–13].

Different treatment protocols and prognoses have been suggested for the clinical stages of CL [11]. The combination of N-methylglucamine antimoniate with Allopurinol is considered the gold standard therapy in CL [11, 14–16]. Clinical response ranges from poor to good, in dependence on the overall initial clinic status of animals and on its individual response to therapy [1–3, 8–11, 17–20].

The critical relevance of host-immune response in CL outcome has been largely demonstrated [1, 11–13, 21]. A complex network of peripheral mechanisms, which are co-evolved to prevent or dampen immune mediated diseases, usually accounts for the activation, expansion and recruitment of T lymphocyte effectors in the infected animals. Regulatory systems include mechanisms intrinsic to the antigen-dependent T cell activation as well as the regulatory suppressor immune-populations, mainly represented by Regulatory T cells (Treg) [22].

Notably, it is conceivable that Treg activity could down-modulate the same inflammatory responses required for infection clearance [22]. During CL, such occurrence may exacerbate the risk that the unbridled parasite growth could lead to a severe disease. However, Treg recruitment is necessary to prevent the onset of severe immune-mediated mechanisms in infected tissues, particularly for the presence of autoimmune processes highly frequent in CL [11, 23–27]. We previously suggested that the increase of cytotoxic T lymphocytes and of Th1 cells together is associated with a reduction of the Treg characterize the CL dogs [28] In addition, Leishmania-specific Treg cells are observed to sites of infection and were described to be dependent on parasite persistence [29]. Notably, the equilibrium between Treg cells and effector lymphocytes appears to control the efficiency of immune responses and disease reactivation [30].

Moreover, it is worth noting that an unbalanced diet and malnutrition could represent primary causes of immune suppression and have been demonstrated to be a major risk factor for the development of visceral Leishmaniosis in humans or animal models [31–34]. Furthermore, the pathways that control immune cell function and metabolism are intimately linked and this relationship might intriguingly provide new strategies to modulate immune functions in several infectious diseases [35, 36].

It is of some relevance that the role of some biological principles - mainly derived from plants and usually referred as nutraceuticals - appears to be of some relevance in modulating the immune system homeostasis [37] as well as their use is of efficacy in dogs [38–41].

In this regard, *Cucumis melo, Aloe vera, Punica granatum, Piper nigrum, Camellia sinensis*, *Ascophyllum nodosum, Grifola frondosa, Glycine max, Echinacea purpurea, Poligonum spp, Carica papaya* and *Curcuma longa* have been described to mediate several immune-modulating effects [42–56]. Anti-oxidant properties have been described for *Cucumis melo, Carica papaya* and *Curcuma longa,* astaxanthin from *Haematococcus pluvialis* as well as for poly-unsaturated fatty acids derived from fish oil. The extracts of *Piper nigrum, Camellia sinensis, Grifola frondosa* and *Glicine max* have been associated with the modulation of inflammatory pathways [57–61]. Punicalagin, the most important active substance contained in the fruit of *Punica granatum*, exerts an immune suppressant activity [47]. Resveratrol, extracted from *Poligonum cuspidatum*, induces a significant reduction in the generation of reactive-oxygen species and can suppress plasma concentrations of pro inflammatory factors like Tumor Necrosis Factor (TNF)-α, Interleukin (IL)-6, and C-reactive protein in humans [62].

In the present study a specific commercial nutraceutical pet food was used as potential immune-modulating diet (IMMD) containing anti-inflammatory and anti-oxidant nutrients and associated with standard anti-*Leishmania* pharmacological treatment in 20 dogs naturally infected by *L. infantum* (IMMD Group). The nutraceutical pet food consisted in a mixed formula of A*scophyllum nodosum, Cucumis melo, Carica papaya, Aloe vera, Astaxanthin* from *Haematococcus pluvialis*, *Curcuma longa, Camellia sinensis, Punica granatum, Piper nigrum, Poligonum spp, Echinacea purpurea*, *Grifola frondosa, Glycine max*, Omega 3 and Omega 6 un-saturated fatty acids from fish oil. As control diet we used a standard diet (SD) based on a commercial pet food without nutraceuticals in 20 CL affected dogs (SD Group).

Since the dogs of SD Groups were enrolled considering the same criteria for the inclusion of those of IMMD Group (see also “Animals and study design” paragraph), the dogs of both groups strongly matched for the starting clinical features except for the type of diet administrated during the study. This approach consents to properly evaluate the potential effects of the immune-modulating diet in a cohort of dogs affected by CL (IMMD Group) in comparison with CL dogs fed with a standard diet (SD Group). Therefore, we evaluated T cell subsets, peripheral blood Treg and Th1 cells at diagnosis and along one-year follow-up in the two groups of sick dogs. To ascertain the degree of changing induced by immune-modulating diet, the results were compared also with the same type of immune-profiles measured in a cohort of 20 sex/age paired healthy dogs.

## Methods

### Ethic statement

This study has been reviewed by Ethical Animal Care and Use Committee of the University of Naples Federico II and received formal Institutional approval (Centro Servizi Veterinari, Università di Napoli FEDERICO II, prot. N. 2015/0071388) in accordance with local and national law, regulations and guidelines. Moreover, the enrolled dogs simply changed the type of food by using commercial products. So, there is no real implementation of an experimental practice to test the effects of a new food, but a mere evaluation of the clinical and functional response of a commercial diet at potential immune-modulating effect in CL. This study avoided discomfort to the animals by the use of proper clinical management. Blood sample collection was cruelty free, without any bloody operation and did not provide for any segregation, even partial, of the animal. In this regards, the study was performed only on household dogs. All enrolled sick animals regularly received the standard therapy for CL.

### The diets

Two groups of dogs (see “Animals and study design” paragraph) were fed two commercial dry pet foods (IMMD and SD) all along the study. An additional group, formed by healthy dogs and analyzed once a time in the trial (see “Animals and study design” paragraph), was fed with standard diets based on the commercial foods to which the animals were already used at the time of the analysis.

All the diets completely fulfill the recommendations for protein, carbohydrate and fat in order to obtain a complete food for a daily ration in dog (as reported in Nutritional Guidelines for complete and complementary pet food for cats and dogs by The European Pet Food Industry Federation). All the foods are commercial and in the form of kibbles industrially produced with extrusion technique.

The IMMD and SD foods, used all along the trial, reported similar analytical composition in nutrients (24 % of crude protein, 12 % of crude oils and fats, 3.7 %, of crude fiber 5 % of crude ash, 9 % of moisture) and, as a consequence, similar Metabolized Energy (ME) of 3.477 kcal/kg corresponding to 14.6 MJ/kg. The IMMD was composed by two mixed components: kibbles, included in the ideal percentage of 93-94 % in weight, and cold-pressed tablets at the 6–7 % in weight of complete food (European patent n. EP 2526781). Overall nutrient profile of the product was obtained by the sum of a first nutrient profile of the kibbles, for feeding purpose, and a second nutrient profile of the tablets for both nutrient and therapeutic purposes. Tablets were composed by 60–80 % of protein hydrolyzed (fish and vegetable ones), 20–40 % of minerals used as glidants and were added by therapeutical substances (*Ascophyllum nodosum, Cucumis melo, Carica papaya, Aloe vera, Astaxanthin from Haematococcus pluvialis, Curcuma longa, Camellia sinensis, Punica granatum, Piper nigrum, Poligonum spp, Echinacea purpurea, Grifola frondosa, Glycine max*, Omega 3 and Omega 6 un-saturated fatty acids from fish, as 1.60 and 1.25 % of oil respectively.

The dry pet food used as SD did not contain the above-mentioned active substances.

The IMMD and SD dietary administration were carefully adjusted during the trial to provide similar caloric animal food intake and to satisfy the nutritional requirement of adult dogs (Table 1). In this regard, the food has been administered twice a day by dog’s owners, according to the specific requirements for the nutrient amount. Owners were instructed to strictly follow the daily dietary tables formulated by veterinarians, according with Manufacturer’s Instructions, as indicated in Table 1.

**Table 1:** Daily table recommendation for diet

Weight (Kg)	Dietary supplement amount per day (g)
1–10	30–180
11–20	190–300
21–35	310–455
36–50	465–595

To guarantee the maintenance energy requirement, food dosage was established using a daily dietary administration (Table 1) based on the equation 130 kcal ME/kg of Body Weight^0.75^ as recommended by the National Research Council Committee on Animal Nutrition, USA. The coefficient used referred to a moderate activity.

In addition, veterinarians have weekly provided and measured the required amount of food to ensure the proper administration to the dogs all along the trial. The IMMD and SD commercial products completely respect the nutritional guidelines established by European Pet Food Industry Federation to provide all nutrients necessary for a canine diet.

### Animals and study design

Forty dogs naturally infected by *L. infantum* (20 males and 20 females, 5–9 years old) from the Campania region (South Italy), which is a CL endemic area, were enrolled with the owner consent. Ten dogs were pure breed (4 German Shepherds, 4 Rottweilers and 2 Labrador Retrievers), while 30 were mongrels (15 females and 15 males, between 20 and 35 kg in weight). The study was performed on household dogs in order to avoid any possible interference dependent on usual environment changing. The pet food was daily administered to the dogs by the owners following the daily dietary tables (see “The diets” paragraph and Table 1). The dogs were under the biweekly supervision of the veterinaries (Department of Veterinary Medicine and Animal Productions, Division of Internal Medicine, University of Naples Federico II, Italy) in order to perform a complete clinical evaluation potentially able to reveal any illness state or nutritional deficiency. The recommendations of the ARRIVE guidelines in animal research were also consulted and considered [63].

Animals were allocated to two groups during the 12 months of the trial. SD Group was composed of 20 dogs treated with meglumine antimoniate (50 mg/kg, subcutaneous, twice daily, for 1 month), allopurinol (10 mg/kg, oral, twice daily, for 6 months) and fed with standard diet. IMMD Group included 20 dogs subjected to the same therapeutic treatment combined with the administration of a diet with potential immune-modulating activity (see also “The diets” paragraph). Animals were equally distributed for breed, sex, weight and clinical signs in the two groups. All the dogs from IMMD and SD Groups fully adapted of new diets. Immune profile evaluation was performed at diagnosis (T0) and after three (T3), six (T6) and twelve (T12) months of trial in all the animals. The full blood count, total proteins, albumin/globulin ratio, urea and creatinine value determinations and immune profile analysis were evaluated at T0, T3, T6 and T12.

The additional control of 20 healthy dogs (12 males, 8 females, 5–7 years old) was analyzed for the same parameters once a time at the beginning of the trial or, in few cases, in the first 15–20 days from the starting evaluation. In this regard, the latter group (Healthy Dogs) provided an useful range of normality for T cell subsets, Treg and Th1 cells in our trial. Indeed, the immune profile evaluations of healthy dogs were used to obtain the control means ± SD for each parameters (platelet count, CD4/CD8 ratio, Treg and INF-γ measurements) to be compared with CL infected dogs of IMMD and SD Groups.

In addition, both indirect fluorescence antibody test (IFAT) and sternal bone marrow aspirate for Leishmania DNA detection by n-PCR were performed at T0 and T12 in IMMD and SD groups and at T0 in healthy dog control (see also “Serological and molecular assays” paragraph).

### Clinical evaluation of dogs

History and clinical examination were performed with the accuracy of guideline criteria for CL diagnosis and classified according to Solano-Gallego et al. [11].

According to AAHA Nutritional Assessment Guidelines for Dogs and Cats, animals were periodically re-examined, for exclusion of signs of food intolerance or allergies related to food or to the enviroment [64]. Thus, pet owners were instructed to evaluate their pets for food intake and appetite, body weight, gastrointestinal signs (feces consistency and entity, vomiting), cutaneous signs (pruritus, dermatitis, poor skin or hair coat), overall appearance and activity. The involved veterinarians supervised all these aspects during the bi-weekly inspection.

No dog received a specific treatment for CL before the enrolment. Criteria for inclusion in this study were the occurrence of clinical signs compatible with Leishmaniosis (weight loss, lethargy, pale mucous membranes, peripheral lymphadenopathy, spleen enlargement, skin lesions, ulcers, ocular signs). Clinical pathological signs including anemia (hematocrit value < 37 %), thrombocytopenia (platelets count < 200 × 103/μL), increase of total proteins (>7,7 g/dL), hyperglobulinemia (albumin/globulin ratio < 0.6), and increase of urea (>50 mg/dL) and creatinine (>1.5 mg/dL) were evaluated. Acute onset of possible arthropod-borne co-infections (such as ehrlichiosis, anaplasmosis or babesiosis) was evaluated and ruled-out all along the study (see also “Diagnostic procedure” paragraph). Dogs were monitored for clinical signs correlated to other illnesses potentially occurring during the trial. Clinical recovery was evaluated at T3, T6 and T12 and was based on the reduction/disappearance of clinical signs listed in the inclusion criteria.

Healthy dogs were enrolled on inclusion criteria that considered the good state of health and the absence of signs related to infectious or metabolic diseases. Furthermore, these dogs were evaluated for the absence of serological indices of infectious diseases (see “Serological and molecular assays” paragraph). History of these dogs revealed that they had not received, in at least the 4 weeks before, any drug therapy can alter the results of the analysis.

None of the dogs belonging to all groups of the study showed signs of food intolerance or allergies related to food or to the enviroment.

At the beginning of the study (T0) and during the follow-up (T3, T6 and T12, for IMMD and SD Groups), the dogs were evaluated for their weight by five-point body condition score (BCS) [65]. Enrolled dogs were of medium body weight of 27 + 2 kg while the BCS, which assesses the nutritional status, ranged from 2.75 to 2.90.

All dogs of the three groups received, as usual, the prophylaxis against infestations by fleas, ticks and mosquitoes by using a monthly local treatment spot-on of specific commercial product. At the beginning of the trial, the dogs were also dewormed with specific commercial product and the treatment was repeated every three months for each dog.

### Blood sample collection

Peripheral blood was collected from the jugular vein into tubes containing ethylene EDTA to perform both the hematological profile and the experimental approach (see also “Diagnostic procedure” paragraph). A complete cell blood count was performed in each sample within 30 min from the collection by a semi-automatic cell counter (Genius S; SEAC Radom Group, Florence, Italy). May-Grünwald-Giemsa-stained blood smears were evaluated for additional confirmation of thrombocytopenia or evidence of platelet clumping. All samples were maintained at room temperature up to 5–6 h prior to processing. In addition, serum aliquots were obtained from the dogs to perform the biochemical profile and serological examination.

The collection of biological samples was practiced in accordance with the national guidelines for animal welfare, only after owner informed consent and on the basis of the received ethical approval (see Ethic Statement paragraph).

### Serological and molecular assays

Detection of anti-Leishmania IgG antibodies was performed by an in-house IFAT assay using L. infantum promastigotes (WHO reference strain MHOM/TN/1980/IPT-1) as antigen and following the protocol recommended by the Office International des Epizooties [66]. The cut-off dilution was set 1:160. A sternal bone marrow aspirate for *Leishmania* DNA detection by nested polymerase chain reaction (n-PCR) was performed. Briefly, the first amplification was carried out in a 50 μl reaction containing 10 μl DNA and 40 μl PCR Master Mix (Promega) with 50 pmol of the kinetoplastid-specific primers R221 and R332 of the small-subunit rRNA gene [67]. For the second amplification, 3 μl of the first PCR product were added to 47 μl of PCR Master Mix (Promega) containing 50 pmol of the *Leishmania*-specific primers R223 and R333 of the same gene [67].

IFAT for *E. canis* was performed using *E. canis* antigen in DH82 cells with a cut-off titer of 1:80. For the *E. canis* n-PCR assay, DNA was extracted from bone marrow aspirate. Briefly, the first PCR was performed in a 25 μl reaction containing 5 μl of DNA template with 12.5 pmol of primer set of universal fD1 and *Ehrlichia* genus-specific EHR16SR [68]. For the second amplification, 5 μl of the first PCR product were added to 20 μl of PCR Master Mix (Promega) containing 12.5 pmol of the *Ehrlichia*-specific primers CANIS and GA1UR of the same gene [68]. The amplification products were analyzed by 1.5 % agarose gel and visualized under UV light.

### Diagnostic procedure

Diagnosis of CL was always confirmed by IFAT and by PCR. Animals with clinical and/or clinical pathological signs attributable to CL, anti-*Leishmania* antibody titers (≥1:160) and positive molecular diagnosis, were included in the study as infected animals (IMMD Group and SD Group). Animals with the absence of clinical signs on physical examination, showing negative IFAT (≤1: 80) and PCR, were considered non-infected and included in the healthy dog control group. Occurrence of infectious and parasitic diseases other than CL was excluded in all dogs. In particular, no evidence of *Ehrlichia canis, Anaplasma phagocytophilum morulae, Babesia canis* trophozoites and microfilariae was observed in peripheral blood smears. Ehrlichiosis was also excluded using IFAT and n-PCR (see also “Serological and molecular assays” paragraph). Finally, *Dirofilaria immitis* infection was ruled out using the Snap Canine Combo Heartworm Antigen Antibody Test (IDEXX).

In addition, the intestinal parasitic infections were excluded in all dogs, before the enrolment. Feces were collected, stored at +4 °C and examined within 48 h. Macroscopic examination was firstly performed for the detection of proglottids of cestodes. Subsequently, each fecal sample was divided into two aliquots. In order to detect common parasite eggs and oocysts, one aliquot was subjected to microscopic analysis by centrifugation-flotation technique with sucrose and sodium nitrate solution (specific gravity: 1360). The second aliquot was used to detect *Giardia* cysts using the SNAP Giardia Test Kit (Idexx Laboratories). All samples tested were negative for internal parasites in all the enrolled dogs.

### Monoclonal antibodies, immunofluorescence, flow cytometry and cell culture

Peripheral blood was employed to evaluate the level of CD3 + CD4+, CD3 + CD8+ T cells and CD3 + CD4 + Foxp3+ Treg cells [22] by immune-fluorescence technique and flow cytometry analysis, as previously described [60]. FITC, Phycoerythrin (PE), Cy-chrome and Allophycocyanin (APC) labeled monoclonal antibodies (mAbs) against dog CD3 (Clone CA17.2A12), CD4 (Clone YKIX302.9), CD8 (Clone YCATE55.9), CD45 (clone CA12.10C12), IFN-γ (Clone CC302), IL-4 (Clone CC303) and isotype-matched controls were purchased from Serotec Ltd (London, UK). Intracellular detection of Foxp3 was performed using a cross-reactive murine FoxP3 antibody (Clone FJK-16 s, eBioscience, San Diego, CA) and the permeabilization buffers provided by the detection Kit (FoxP3 Staining Set, eBioscience), as described [60, 61]. The detection of Treg was based on the CD3+ CD4+ and Foxp3 staining FACS strategy, as described [28, 69]. CD8+ and CD4+ T cell subsets were always identified by a combination of canine specific anti-CD3 together with anti-CD4 or anti-CD8 mAbs. To analyze the production of IFN-γ, purified PBMC were cultured overnight in presence of PMA and Ionomycin (Sigma-Aldrich, St. Louis, MO). This approach has been widely indicated for the study of cytokine profile in human and animal models [70, 71]. Intracellular staining with the mAbs recognizing dog IFN-γ or isotype-matched controls (Serotec) was performed by a fixing/permeabilization kit (Caltag, Burlingame, CA). To avoid extra cellular cytokine export, the cell cultures were incubated in the presence of 5 μg/ml of Brefeldin-A (Sigma-Aldrich, St. Louis, MO), as described [62].

Cells were cultured in RPMI 1640 (Biochrom K.G., Berlin, Germany) supplemented with 5 % heat inactivated fetal bovine serum and 2 mM glutamine (Biochrom) at 37 °C in 5 % CO2/95 % air. All phenotypes referred to flow cytometry analysis of the lymphocyte population gated by using Forward Scatter (FSC) and Side Scatter (SSC) parameters. Flow cytometry and data analysis were performed by using a two laser equipped *FACScalibur* apparatus and the CellQuest analysis software (Becton Dickinson, Mountain View, CA).

### Statistical analysis

The statistical analysis was performed by the one way Analysis of Variance (ANOVA) with the post-test corrections according with Tukey-Kramer Test for Multiple Comparisons using *GraphPad Prism* Software (GraphPad Prism Inc, San Diego, CA, USA). Results were considered significant at *p* < 0.05.

## Results

### Clinical evaluation

Enrolled sick dogs were symptomatic. Clinical signs were lymphadenopathy in 23 dogs of 40 (63 %), dermatitis in 13 dogs of 40 (33 %), alopecia in 10 dogs of 40 (25 %) and splenomegaly in 20 dogs of 40 (50 %). A progressive improvement of clinical conditions was evident in sick dogs along the 12 months of follow-up. No significant increment in dog body weight and in BCS was detected at T3, T6 and T12 when compared to T0, regardless to diet. PCR detection of the parasite DNA remained steadily positive in all sick dogs during the study. None dog evidenced allergies or food-intolerances during the trial. No enrolled dog died during the trial.

The clinical recovery was evaluated considering the disappearance of clinical signs at the end of the trial an only basing on an on-site clinical observation. Therefore, a statistical analysis was not performed for the clinical recovery. Nevertheless, it could be of some relevance that the disappearance of clinical signs was observed in 10 of 20 dogs (50 %) of the SD Group and in 13 of 20 dogs (65 %) of the IMMD Group.

**Fig. 1:**
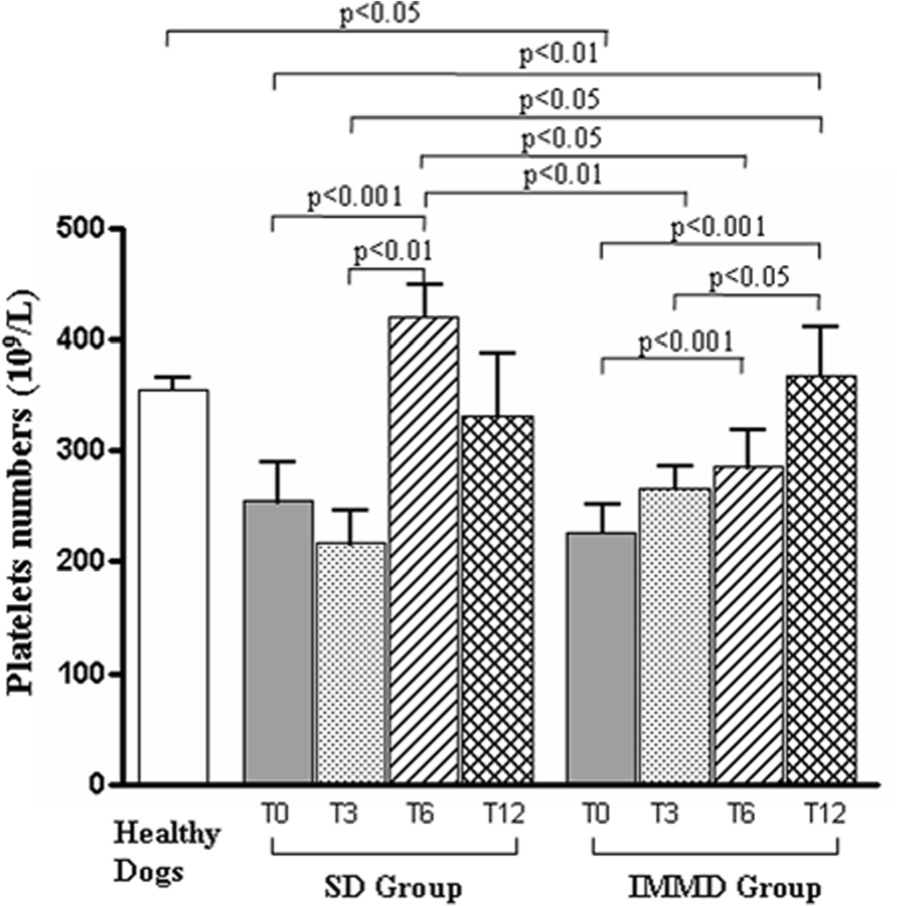
Analysis of platelet number. Values indicate the platelet number in healthy dogs, in sick dogs at T0, T3, T6 and T12 in SD Group (pharmacological treatment alone) and IMMD Group (pharmacological treatment and immune-modulating diet), as indicated. The column of Healthy Dogs refers to the means ± SD values of normal range (see material and methods) of platelet count. Results were considered significant at *p* < 0.05

In addition, all sick dogs showed a significant decrease of platelet number at diagnosis (T0) in comparison with healthy dogs (Fig. 1). This alteration completely disappeared at T12, regardless the group assignment. A strong increase of platelets in SD Group dogs was observed at T6, with a slight decrease at T12 but still remaining in the normal range, since it was not statistically significant if compared with healthy dog controls. IMMD Group dogs showed a significant increase in platelet number becoming similar to healthy controls at T6 and T12.

Comparative analysis of biochemical and hematological parameters between SD Group and IMMD Group dogs was performed at diagnosis (T0) and at T3, T6 and at T12. Increase in the total protein amount and globulin was observed in sick dogs. A/G ratio analysis showed no significant changes in CL dogs, regardless the group assignment (not shown). Albumin levels and creatinine values remained within the normal limits in all dogs during the study.

Intriguingly, hemoglobin evaluation showed a mean increase of more than 1.5 g/L in all CL dogs at T6 and T12, regardless the group assignment as compared with the T0 values (not shown).

No significant difference was observed along the follow-up in the number of white blood cells in the CL dogs, regardless the group assignment (data not shown).

### Immune phenotype analysis

**Fig. 2:**
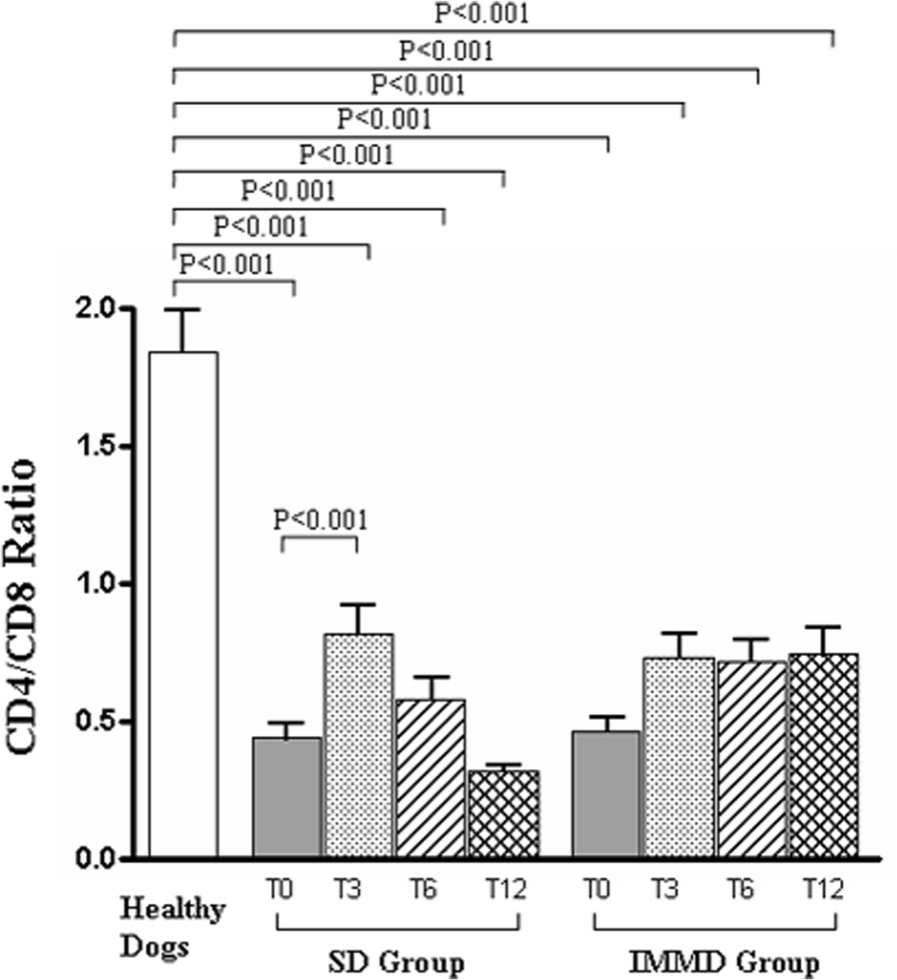
The CD4/CD8 ratio reduction is observed in CL sick dogs, regardless the diet. Values indicate the CD4/CD8 ratio observed in healthy dogs, in sick dogs at T0, T3, T6 and T12 in SD Group (pharmacological treatment alone) and IMMD Group (pharmacological treatment and immune-modulating diet), as indicated. The column of Healthy Dogs refers to the means ± SD values of normal range (see material and methods) of CD4/CD8 ratio in healthy controls. Results were considered significant at *p* < 0.05

We analyzed CD4/CD8 ratio in dogs along the 12-months of follow-up. As indicated in Fig. 2, we confirmed the significant decrease of CD4/CD8 ratio at T0 in CL dogs when compared to healthy dogs, as by us already described [60]. The observed increase of CD8+ T cells explains the decrease of CD4/CD8 ratio. This alteration remained substantially unmodified along the follow up in both SD and IMMD Groups. Indeed, sick dogs maintained a significant reduction of CD4/CD8 ratio in comparison to control animals at all the time points.

**Fig. 3:**
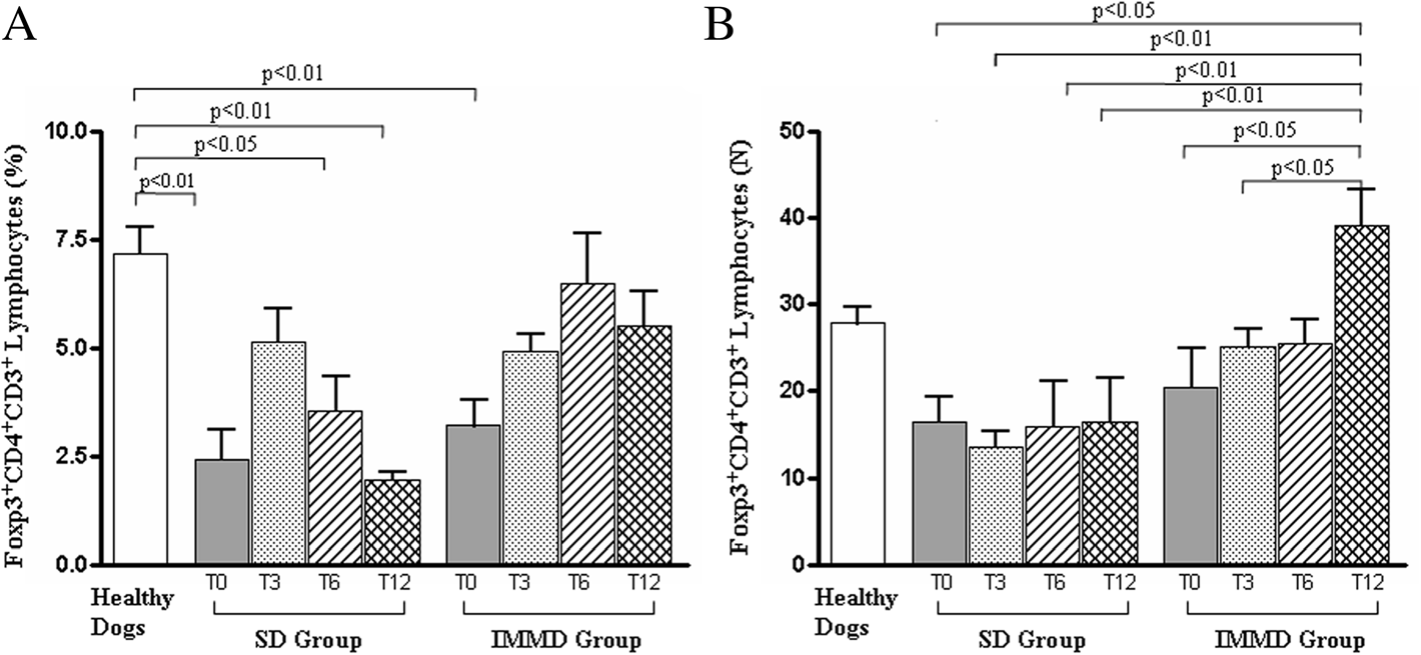
The combination of the pharmacological treatment with an immune-modulating diet restores Treg percentage and count in sick dogs. Panel A and B show the percentage and count (x10^−6^/L), respectively, of Foxp3 + CD4 + CD3+ (Treg) cells in healthy dogs, in sick dogs at T0, T3, T6 and T12 in SD Group (pharmacological treatment alone) and IMMD Group (pharmacological treatment and immune-modulating diet), as indicated. The column of Healthy Dogs refers to the means ± SD values of normal range (see material and methods) of Treg measurements in healthy controls. Results were considered significant at *p* < 0.05

Moreover, when the analysis specifically focused on CD4/CD8 ratio in the SD Group and IMMD Group, significant differences between the groups were observed along the follow-up. As shown, an increase of CD4/CD8 ratio was revealed in SD Group at T3 (0.81 ± 0.09; *p* < 0.001), while at T6 (0.58 ± 0.08) and at T12 (0.31 ± 0.02) the values substantially resembled to those observed at T0 (0.43 ± 0.05). At variance, the trend of increase in CD4/CD8 ratio at T3 (0.73 ± 0.08 versus 0.46 ± 0.04) was maintained at T6 (0.71 ± 0.08) and T12 (0.74 ± 0.09) in IMMD Group. Notably, these values remained steadily and significantly lower than in healthy dogs (Fig. 2). As shown in Fig. 3, a significant reduction in the percentage of Treg of all sick dogs was observed at T0. This result confirms our previous observation on Treg levels in CL [28]. A slight recovery of Treg percentage was observed only at T3 in SD Group, while this effect disappeared at T6 and T12. In contrast, it is worth noting that percentage of Treg became similar to healthy animals in IMDD Group at T3, T6 and T12 (Fig. 3). Indeed, the differences between the Treg percentages between healthy and IMMD Group dogs substantially disappeared.

This result strongly suggests that the occurrence of a quite stable recovery of Treg was correlated to the immune-modulating diet administration.

In addition, anti-*Leishmania* treatment alone was unable to modify Treg level in CL. At variance, the combination of drug with the potential immune-modulating diet seems to be associated with a significant increase of Treg population that reaches normal values in IMMD Group.

**Fig. 4:**
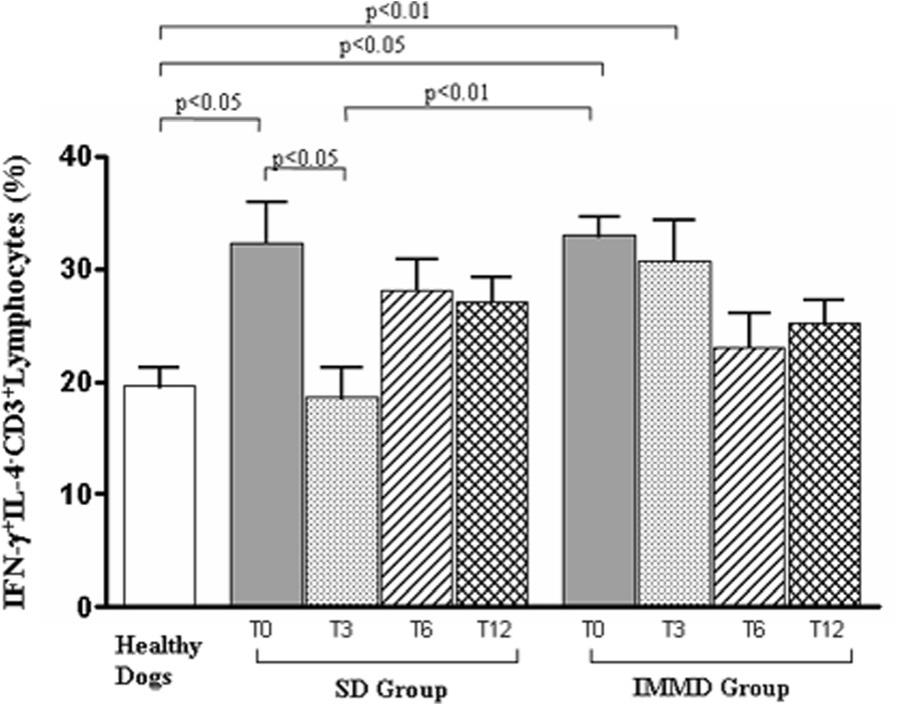
Association of the pharmacological treatment with an immune-modulating diet modulates the presence of IFN-γ^+^ IL^−^4-CD3^+^ T lymphocytes (Th1) in sick dogs. Values indicated the percentage of Th1 lymphocytes in healthy dogs, in sick dogs at T0, T3, T6 and T12 in SD Group (pharmacological treatment alone) and IMMD Group (pharmacological treatment and immune-modulating diet), as indicated. The column of Healthy Dogs refers to the means ± SD values of normal range (see material and methods) of Th1 measurement in healthy controls. Results were considered significant at *p* < 0.05

Moreover, we asked if anti-*Leishmania* therapy alone and/or associated with diet modification could affect Th1 activity in CL. As shown in Fig. 4, sick dogs showed a significant increase of Th1 cells at T0 as compared with healthy dogs, regardless the group assignment. This result is in accordance with our previous data on CL [28]. The comparative analysis of sick dogs with healthy controls revealed the occurrence of a significant decrease of Th1 cells from T3 to T12 in SD Group, although a trend of increase was observed at T6 and T12. At variance, IMMD Group dogs showed a progressive decrease of Th1 cells, whose levels became similar to healthy controls at T6 and T12.

## Discussion

We analyzed the peripheral lymphocyte subsets, in particular the Treg and Th1 cells, in 20 CL dogs treated with standard anti-*Leishmania* pharmacological therapy combined to standard diet (SD Group) and in 20 CL dogs treated with anti-*Leishmania* therapy and the immune-modulating diet (IMMD Group). The dogs of two groups were enrolled considering the same inclusion criteria so that they strongly matched for the starting clinical features except for the type of diet administrated during the study. The trial was performed on household dogs in order to avoid any possible interference dependent on usual environment changing and pet food was daily administered to the dogs by the owners following the daily dietary tables (Table 1). The two diets provided similar caloric animal food intake and satisfied the nutritional requirement of adult dogs.

Therefore, it is highly conceivable that the only noticeable difference between the two diets is the presence of nutraceuticals in the pet food administered to IMMD Group dogs.

In light of this, our study suggested that a diet supplemented with *Ascophyllum nodosum, Cucumis melo, Carica papaya, Aloe vera, Astaxanthin from Haematococcus pluvialis, Curcuma longa, Camellia sinensis, Punica granatum, Piper nigrum, Poligonum spp, Echinacea purpurea, Grifola frondosa, Glycine max*, Omega 3 and Omega 6 un-saturated fatty acids from fish oil induced an intriguing immune-modulation in CL dogs undergoing 12 months of treatment with standard pharmacological therapy (IMMD Group).

In particular, we observed that the significant reduction of Treg subset, frequently associated with chronic CL [28], was significantly restored by an immune-modulating diet administration if compared to SD Group dogs and when considering the “normal value” (see Animals and study design paragraph) obtained by Treg measurements in healthy dog controls. Such modification was maintained in a one-year follow-up. Notably, IMMD Group dogs also showed a progressive and significant decrease of Th1 cells, whose levels became similar to healthy dogs at T6 and T12.

Therefore, anti-*Leishmania* treatment combined with specific nutraceutical diet, supplemented by nutrients selected for their potential immune-modulating properties and rich in essential fatty acids, was associated with significant changes in immune profile of sick dogs.

In this regard, it is of note that the nutraceuticals used in this study were previously suggested as antioxidants and immune-modulating substances to reach the physiological status in several models of disease in human [43, 62] and animals [49, 51, 54, 55, 61].

The effect of nutraceutical diet appeared to be specific for Treg and Th1 lymphocytes, since other type of T cells were substantially unaffected. Indeed, CL pharmacological treatment alone or in the presence of specific immune-modulating nutrient supplementation did not alter the CD4/CD8 ratio and did not affect the increase in CD8+ T cell effectors in sick dogs along the follow-up. Notably, the absence of effect on such T cell effectors appears to be a favorable effect. Indeed, it is conceivable that the maintenance of a high percentage of T cytotoxic effectors could foster an effective immune response against the parasite and it is mainly correlated to the persistence of this chronic infection in the animals. Moreover, the increase in Treg percentage could have a role in reducing the immune-mediated damages to the tissues frequently associated with CL. The decrease in Th1 inflammatory response may sustain this hypothesis.

In this regard, both the clinical outcome of the disease and the occurrence of immune-pathological complications have been largely associated with the anti-*Leishmania* immune response orchestration: i.e. murine cutaneous Leishmaniosis model demonstrated that Th1 and Th2 responses are in counter-regulatory dependence [7].

It is of note that the pharmacological-treated dogs fed with IMMD dietary regimen recovered their condition, only basing on clinical observation, in a higher percentage if compared with those animals maintained with standard diet (65 % vs. 50 %) at the end of the study. In addition, the dogs treated with the combination of standard therapy and the IMMD showed a significant increase in platelet number along the study.

## Conclusions

 Our data evidenced as the pharmacological treatment alone was unable to induce long lasting changes in pro-inflammatory response and to modulate Treg in sick dogs, while the combination with immune-modulating diet was associated with a significant restoration of Treg level and with the decrease in Th1 inflammatory response.

Despite the effects on Treg and Th1 cells do not correlate with fully changes in clinical outcome of infection, it is of some relevance that the increase of Treg and the modulation of Th1 inflammatory response could have a role in reducing the immune-pathological injury resulting from CL disease.

In this context, the possibility that the occurrence of a mild inflammatory context determined by increased Treg level could ameliorate immune-mediated pathological effects, as the immune-mediated thrombocytopenia [28], appears to be of some relevance.

Although further investigations are certainly needed on the molecular mechanisms of these findings, to the best of our knowledge this research represents one of the few in vivo studies on the diet effects in modulating the immune responses during infectious disease. This study could open an interesting scenario on the role of diet in modulating the immune response and to design novel combined treatment against infectious disease for dogs and, in perspective, for humans.

### Study limitations

Notably, this research has some study limitations:

- the absence of control groups of dogs fed with other anti-oxidant diet, able to verify the immune-modulating properties of other extracts of plants different from those employed in the present research, represents the main relevant limitation;
- The use of a specific breed of dog, instead of a mixed group of race, could highlight peculiarity in disease susceptibility and in diet effects that may be been unobserved in the use of a heterogeneous group. At the same time, the here used heterogeneous cohort of dogs could have the merit to reveal general effects not segregated on race specificity;
- Skin biopsy has been not performed to ascertain the relevance for the parasitic spread in skin;
- Fecal examination has been not performed during trial to rule out any possible interference of worms in some dogs.
- None blinded study was conducted. Dog owners, veterinarians and researchers involved in the study were aware of the type of diet administered.

## Abbreviations

CL: Canine Leishamiasis
Treg: CD3+ CD4+ Foxp3+ Regulatory T cells
Th1: CD3+ CD4+ IFN-γ + T helper 1
IMMD: Immune-modulating diet
SD: Standard diet
